# Supplementary material for: Fourteen-Day Evolution of COVID-19 Symptoms during the Third Wave in Nonvaccinated Subjects and Effects of Hesperidin Therapy: A Randomized, Double-Blinded, Placebo-Controlled Study
Source: Evid Based Complement Alternat Med. 2022 Nov 3;2022:3125662. doi: 10.1155/2022/3125662 (PMC9649310; doi:10.1155/2022/3125662)
Supplement: Supplementary Materials — S1 Protocol. Version 2.0 of the Hesperidin study protocol approved by the Montreal Heart Institute Research and Ethics Committees. S2 table. Proportion of patients with group A COVID-19 symptoms,attrition bias analysis. S3 table. Summary and statistical analysis for the presence of each 13 COVID-19 symptoms. S4 table. Overall summary of treatment emergent adverse events (TEAEs) and serious adverse events (TESAEs). [file 3125662.f1.zip › S3 tables.pdf]

2 - Efficacy Analysis
2.2 - Secondary Analysis
Table 2.2.3a - Summary of and Statistical Analysis for Presence of Cough at Day 1 to 14, ITT Population

		Placebo N=109	Hesperidin N=107	All N=216
DAY 1	n	107	105	212
	No	40 (37.4%)	43 (41.0%)	83 (39.2%)
	Yes	67 (62.6%)	62 (59.0%)	129 (60.8%)
DAY 2	n	106	105	211
	No	44 (41.5%)	43 (41.0%)	87 (41.2%)
	Yes	62 (58.5%)	62 (59.0%)	124 (58.8%)
DAY 3	n	103	102	205
	No	49 (47.6%)	48 (47.1%)	97 (47.3%)
	Yes	54 (52.4%)	54 (52.9%)	108 (52.7%)
DAY 4	n	103	96	199
	No	46 (44.7%)	47 (49.0%)	93 (46.7%)
	Yes	57 (55.3%)	49 (51.0%)	106 (53.3%)
DAY 5	n	102	95	197
	No	53 (52.0%)	52 (54.7%)	105 (53.3%)
	Yes	49 (48.0%)	43 (45.3%)	92 (46.7%)
DAY 6	n	103	92	195
	No	55 (53.4%)	54 (58.7%)	109 (55.9%)
	Yes	48 (46.6%)	38 (41.3%)	86 (44.1%)
DAY 7	n	102	91	193
	No	57 (55.9%)	54 (59.3%)	111 (57.5%)
	Yes	45 (44.1%)	37 (40.7%)	82 (42.5%)
DAY 8	n	101	90	191
	No	64 (63.4%)	58 (64.4%)	122 (63.9%)
	Yes	37 (36.6%)	32 (35.6%)	69 (36.1%)
DAY 9	n	100	89	189
	No	61 (61.0%)	62 (69.7%)	123 (65.1%)
	Yes	39 (39.0%)	27 (30.3%)	66 (34.9%)

Note:
Comparaisons at day 3,7,10 and 14 are considered for secondary analysis. The other days are used for exploratory analysis.

CONFIDENTIALITY STATEMENT: Information contained in this statistical report is confidential and should not be disclosed to other parties than those directly involved with the execution of this study without written authorization from Montreal Heart Institute

2 - Efficacy Analysis
2.2 - Secondary Analysis
Table 2.2.3a - Summary of and Statistical Analysis for Presence of Cough at Day 1 to 14, ITT Population

		Placebo N=109	Hesperidin N=107	All N=216
DAY 10	n	100	90	190
	No	65 (65.0%)	64 (71.1%)	129 (67.9%)
	Yes	35 (35.0%)	26 (28.9%)	61 (32.1%)
DAY 11	n	98	86	184
	No	66 (67.3%)	64 (74.4%)	130 (70.7%)
	Yes	32 (32.7%)	22 (25.6%)	54 (29.3%)
DAY 12	n	99	85	184
	No	68 (68.7%)	62 (72.9%)	130 (70.7%)
	Yes	31 (31.3%)	23 (27.1%)	54 (29.3%)
DAY 13	n	98	83	181
	No	67 (68.4%)	64 (77.1%)	131 (72.4%)
	Yes	31 (31.6%)	19 (22.9%)	50 (27.6%)
DAY 14	n	95	79	174
	No	66 (69.5%)	58 (73.4%)	124 (71.3%)
	Yes	29 (30.5%)	21 (26.6%)	50 (28.7%)

Note:
Comparaisons at day 3,7,10 and 14 are considered for secondary analysis. The other days are used for exploratory analysis.

CONFIDENTIALITY STATEMENT: Information contained in this statistical report is confidential and should not be disclosed to other parties than those directly involved with the execution of this study without written authorization from Montreal Heart Institute

2 – Efficacy Analysis
2.2 – Secondary Analysis
Table 2.2.3a - Summary of and Statistical Analysis for Presence of Cough at Day 1 to 14, ITT Population (continued)

Generalized linear mixed model (repeated binary logistic regression) with terms for treatment group, time and treatment group x time interaction

Contrast	OR (95% CI)	P-value
Hesperidin vs Placebo at Day 3	1.02 (0.59; 1.77)	0.9416
Hesperidin vs Placebo at Day 7	0.87 (0.49; 1.55)	0.6296
Hesperidin vs Placebo at Day 10	0.75 (0.41; 1.40)	0.3711
Hesperidin vs Placebo at Day 14	0.82 (0.42; 1.61)	0.5696

CONFIDENTIALITY STATEMENT: Information contained in this statistical report is confidential and should not be disclosed to other parties than those directly involved with the execution of this study without written authorization from Montreal Heart Institute

2 – Efficacy Analysis
2.2 – Secondary Analysis
Table 2.2.3b – Summary of and Statistical Analysis for Presence of Fever at Day 1 to 14, ITT Population

		Placebo N=109	Hesperidin N=107	All N=216
DAY 1	n	105	102	207
	No	100 (95.2%)	99 (97.1%)	199 (96.1%)
	Yes	5 (4.8%)	3 (2.9%)	8 (3.9%)
DAY 2	n	105	103	208
	No	101 (96.2%)	100 (97.1%)	201 (96.6%)
	Yes	4 (3.8%)	3 (2.9%)	7 (3.4%)
DAY 3	n	102	101	203
	No	96 (94.1%)	96 (95.0%)	192 (94.6%)
	Yes	6 (5.9%)	5 (5.0%)	11 (5.4%)
DAY 4	n	102	95	197
	No	99 (97.1%)	92 (96.8%)	191 (97.0%)
	Yes	3 (2.9%)	3 (3.2%)	6 (3.0%)
DAY 5	n	102	94	196
	No	98 (96.1%)	89 (94.7%)	187 (95.4%)
	Yes	4 (3.9%)	5 (5.3%)	9 (4.6%)
DAY 6	n	102	91	193
	No	99 (97.1%)	88 (96.7%)	187 (96.9%)
	Yes	3 (2.9%)	3 (3.3%)	6 (3.1%)
DAY 7	n	101	90	191
	No	97 (96.0%)	87 (96.7%)	184 (96.3%)
	Yes	4 (4.0%)	3 (3.3%)	7 (3.7%)
DAY 8	n	100	90	190
	No	97 (97.0%)	87 (96.7%)	184 (96.8%)
	Yes	3 (3.0%)	3 (3.3%)	6 (3.2%)
DAY 9	n	99	89	188
	No	98 (99.0%)	87 (97.8%)	185 (98.4%)
	Yes	1 (1.0%)	2 (2.2%)	3 (1.6%)

Note:
Comparaisons at day 3,7,10 and 14 are considered for secondary analysis. The other days are used for exploratory analysis.

CONFIDENTIALITY STATEMENT: Information contained in this statistical report is confidential and should not be disclosed to other parties than those directly involved with the execution of this study without written authorization from Montreal Heart Institute

2 - Efficacy Analysis
2.2 - Secondary Analysis
Table 2.2.3b - Summary of and Statistical Analysis for Presence of Fever at Day 1 to 14, ITT Population

		Placebo N=109	Hesperidin N=107	All N=216
DAY 10	n	99	90	189
	No	97 (98.0%)	87 (96.7%)	184 (97.4%)
	Yes	2 (2.0%)	3 (3.3%)	5 (2.6%)
DAY 11	n	97	86	183
	No	96 (99.0%)	84 (97.7%)	180 (98.4%)
	Yes	1 (1.0%)	2 (2.3%)	3 (1.6%)
DAY 12	n	98	85	183
	No	97 (99.0%)	84 (98.8%)	181 (98.9%)
	Yes	1 (1.0%)	1 (1.2%)	2 (1.1%)
DAY 13	n	97	83	180
	No	96 (99.0%)	82 (98.8%)	178 (98.9%)
	Yes	1 (1.0%)	1 (1.2%)	2 (1.1%)
DAY 14	n	94	79	173
	No	93 (98.9%)	79 (100.0%)	172 (99.4%)
	Yes	1 (1.1%)	0 (0.0%)	1 (0.6%)

Note:
Comparaisons at day 3,7,10 and 14 are considered for secondary analysis. The other days are used for exploratory analysis.

CONFIDENTIALITY STATEMENT: Information contained in this statistical report is confidential and should not be disclosed to other parties than those directly involved with the execution of this study without written authorization from Montreal Heart Institute

2 – Efficacy Analysis
2.2 – Secondary Analysis
Table 2.2.3b - Summary of and Statistical Analysis for Presence of Fever at Day 1 to 14, ITT Population (continued)

Generalized linear mixed model (repeated binary logistic regression) with terms for treatment group, time and treatment group x time interaction

Contrast	OR (95% CI)	P-value
Hesperidin vs Placebo at Day 3	0.83 (0.26; 2.67)	0.7583
Hesperidin vs Placebo at Day 7	0.84 (0.20; 3.58)	0.8091
Hesperidin vs Placebo at Day 10	1.67 (0.30; 9.42)	0.5591
Hesperidin vs Placebo at Day 14	0.00 (0.00; ND)	0.9983

CONFIDENTIALITY STATEMENT: Information contained in this statistical report is confidential and should not be disclosed to other parties than those directly involved with the execution of this study without written authorization from Montreal Heart Institute

2 - Efficacy Analysis
2.2 - Secondary Analysis
Table 2.2.3c - Summary of and Statistical Analysis for Presence of Feverish or Chills at Day 1 to 14, ITT population

		Placebo N=109	Hesperidin N=107	All N=216
DAY 1	n	107	105	212
	No	75 (70.1%)	69 (65.7%)	144 (67.9%)
	Yes	32 (29.9%)	36 (34.3%)	68 (32.1%)
DAY 2	n	106	105	211
	No	82 (77.4%)	70 (66.7%)	152 (72.0%)
	Yes	24 (22.6%)	35 (33.3%)	59 (28.0%)
DAY 3	n	103	102	205
	No	82 (79.6%)	78 (76.5%)	160 (78.0%)
	Yes	21 (20.4%)	24 (23.5%)	45 (22.0%)
DAY 4	n	103	96	199
	No	89 (86.4%)	77 (80.2%)	166 (83.4%)
	Yes	14 (13.6%)	19 (19.8%)	33 (16.6%)
DAY 5	n	102	95	197
	No	89 (87.3%)	79 (83.2%)	168 (85.3%)
	Yes	13 (12.7%)	16 (16.8%)	29 (14.7%)
DAY 6	n	103	92	195
	No	93 (90.3%)	79 (85.9%)	172 (88.2%)
	Yes	10 (9.7%)	13 (14.1%)	23 (11.8%)
DAY 7	n	102	91	193
	No	92 (90.2%)	81 (89.0%)	173 (89.6%)
	Yes	10 (9.8%)	10 (11.0%)	20 (10.4%)
DAY 8	n	101	90	191
	No	91 (90.1%)	82 (91.1%)	173 (90.6%)
	Yes	10 (9.9%)	8 (8.9%)	18 (9.4%)
DAY 9	n	100	89	189
	No	93 (93.0%)	85 (95.5%)	178 (94.2%)
	Yes	7 (7.0%)	4 (4.5%)	11 (5.8%)

Note:
Comparaisons at day 3,7,10 and 14 are considered for secondary analysis. The other days are used for exploratory analysis.

CONFIDENTIALITY STATEMENT: Information contained in this statistical report is confidential and should not be disclosed to other parties than those directly involved with the execution of this study without written authorization from Montreal Heart Institute

2 - Efficacy Analysis
2.2 - Secondary Analysis
Table 2.2.3c - Summary of and Statistical Analysis for Presence of Feverish or Chills at Day 1 to 14, ITT population

		Placebo N=109	Hesperidin N=107	All N=216
DAY 10	n	100	90	190
	No	94 (94.0%)	86 (95.6%)	180 (94.7%)
	Yes	6 (6.0%)	4 (4.4%)	10 (5.3%)
DAY 11	n	98	86	184
	No	93 (94.9%)	83 (96.5%)	176 (95.7%)
	Yes	5 (5.1%)	3 (3.5%)	8 (4.3%)
DAY 12	n	99	85	184
	No	95 (96.0%)	83 (97.6%)	178 (96.7%)
	Yes	4 (4.0%)	2 (2.4%)	6 (3.3%)
DAY 13	n	98	83	181
	No	96 (98.0%)	82 (98.8%)	178 (98.3%)
	Yes	2 (2.0%)	1 (1.2%)	3 (1.7%)
DAY 14	n	95	79	174
	No	92 (96.8%)	78 (98.7%)	170 (97.7%)
	Yes	3 (3.2%)	1 (1.3%)	4 (2.3%)

Note:
Comparaisons at day 3,7,10 and 14 are considered for secondary analysis. The other days are used for exploratory analysis.

CONFIDENTIALITY STATEMENT: Information contained in this statistical report is confidential and should not be disclosed to other parties than those directly involved with the execution of this study without written authorization from Montreal Heart Institute

2 – Efficacy Analysis
2.2 – Secondary Analysis
Table 2.2.3c - Summary of and Statistical Analysis for Presence of Feverish or Chills at Day 1 to 14, ITT Population (continued)

Generalized linear mixed model (repeated binary logistic regression) with terms for treatment group, time and treatment group x time interaction

Contrast	OR (95% CI)	P-value
Hesperidin vs Placebo at Day 3	1.20 (0.62; 2.34)	0.5894
Hesperidin vs Placebo at Day 7	1.14 (0.45; 2.89)	0.7887
Hesperidin vs Placebo at Day 10	0.73 (0.20; 2.70)	0.6348
Hesperidin vs Placebo at Day 14	0.39 (0.04; 3.92)	0.4257

CONFIDENTIALITY STATEMENT: Information contained in this statistical report is confidential and should not be disclosed to other parties than those directly involved with the execution of this study without written authorization from Montreal Heart Institute

2 - Efficacy Analysis
2.2 - Secondary Analysis
Table 2.2.3d - Summary of and Statistical Analysis for Presence of Sore Throat at Day 1 to 14, ITT population

		Placebo N=109	Hesperidin N=107	All N=216
DAY 1	n	107	105	212
	No	66 (61.7%)	62 (59.0%)	128 (60.4%)
	Yes	41 (38.3%)	43 (41.0%)	84 (39.6%)
DAY 2	n	106	105	211
	No	71 (67.0%)	65 (61.9%)	136 (64.5%)
	Yes	35 (33.0%)	40 (38.1%)	75 (35.5%)
DAY 3	n	103	102	205
	No	80 (77.7%)	69 (67.6%)	149 (72.7%)
	Yes	23 (22.3%)	33 (32.4%)	56 (27.3%)
DAY 4	n	103	96	199
	No	80 (77.7%)	69 (71.9%)	149 (74.9%)
	Yes	23 (22.3%)	27 (28.1%)	50 (25.1%)
DAY 5	n	102	95	197
	No	79 (77.5%)	70 (73.7%)	149 (75.6%)
	Yes	23 (22.5%)	25 (26.3%)	48 (24.4%)
DAY 6	n	103	92	195
	No	86 (83.5%)	76 (82.6%)	162 (83.1%)
	Yes	17 (16.5%)	16 (17.4%)	33 (16.9%)
DAY 7	n	102	91	193
	No	89 (87.3%)	74 (81.3%)	163 (84.5%)
	Yes	13 (12.7%)	17 (18.7%)	30 (15.5%)
DAY 8	n	101	90	191
	No	88 (87.1%)	78 (86.7%)	166 (86.9%)
	Yes	13 (12.9%)	12 (13.3%)	25 (13.1%)
DAY 9	n	100	89	189
	No	87 (87.0%)	84 (94.4%)	171 (90.5%)
	Yes	13 (13.0%)	5 (5.6%)	18 (9.5%)

Note:
Comparaisons at day 3,7,10 and 14 are considered for secondary analysis. The other days are used for exploratory analysis.

CONFIDENTIALITY STATEMENT: Information contained in this statistical report is confidential and should not be disclosed to other parties than those directly involved with the execution of this study without written authorization from Montreal Heart Institute

2 - Efficacy Analysis
2.2 - Secondary Analysis
Table 2.2.3d - Summary of and Statistical Analysis for Presence of Sore Throat at Day 1 to 14, ITT population

		Placebo N=109	Hesperidin N=107	All N=216
DAY 10	n	100	90	190
	No	92 (92.0%)	84 (93.3%)	176 (92.6%)
	Yes	8 (8.0%)	6 (6.7%)	14 (7.4%)
DAY 11	n	98	86	184
	No	90 (91.8%)	82 (95.3%)	172 (93.5%)
	Yes	8 (8.2%)	4 (4.7%)	12 (6.5%)
DAY 12	n	99	85	184
	No	93 (93.9%)	81 (95.3%)	174 (94.6%)
	Yes	6 (6.1%)	4 (4.7%)	10 (5.4%)
DAY 13	n	98	83	181
	No	93 (94.9%)	81 (97.6%)	174 (96.1%)
	Yes	5 (5.1%)	2 (2.4%)	7 (3.9%)
DAY 14	n	95	79	174
	No	91 (95.8%)	76 (96.2%)	167 (96.0%)
	Yes	4 (4.2%)	3 (3.8%)	7 (4.0%)

Note:
Comparaisons at day 3,7,10 and 14 are considered for secondary analysis. The other days are used for exploratory analysis.

CONFIDENTIALITY STATEMENT: Information contained in this statistical report is confidential and should not be disclosed to other parties than those directly involved with the execution of this study without written authorization from Montreal Heart Institute

2 – Efficacy Analysis
2.2 – Secondary Analysis
Table 2.2.3d - Summary of and Statistical Analysis for Presence of Sore Throat at Day 1 to 14, ITT Population (continued)

Generalized linear mixed model (repeated binary logistic regression) with terms for treatment group, time and treatment group x time interaction

Contrast	OR (95% CI)	P-value
Hesperidin vs Placebo at Day 3	1.66 (0.89; 3.11)	0.1113
Hesperidin vs Placebo at Day 7	1.57 (0.71; 3.47)	0.2613
Hesperidin vs Placebo at Day 10	0.82 (0.27; 2.49)	0.7273
Hesperidin vs Placebo at Day 14	0.90 (0.19; 4.19)	0.8909

CONFIDENTIALITY STATEMENT: Information contained in this statistical report is confidential and should not be disclosed to other parties than those directly involved with the execution of this study without written authorization from Montreal Heart Institute

2 – Efficacy Analysis
2.2 – Secondary Analysis
Table 2.2.3e – Summary of and Statistical Analysis for Presence of Runny Nose at Day 1 to 14, ITT population

		Placebo N=109	Hesperidin N=107	All N=216
DAY 1	n	107	105	212
	No	57 (53.3%)	52 (49.5%)	109 (51.4%)
	Yes	50 (46.7%)	53 (50.5%)	103 (48.6%)
DAY 2	n	106	105	211
	No	59 (55.7%)	50 (47.6%)	109 (51.7%)
	Yes	47 (44.3%)	55 (52.4%)	102 (48.3%)
DAY 3	n	103	102	205
	No	65 (63.1%)	54 (52.9%)	119 (58.0%)
	Yes	38 (36.9%)	48 (47.1%)	86 (42.0%)
DAY 4	n	103	96	199
	No	68 (66.0%)	56 (58.3%)	124 (62.3%)
	Yes	35 (34.0%)	40 (41.7%)	75 (37.7%)
DAY 5	n	102	95	197
	No	70 (68.6%)	59 (62.1%)	129 (65.5%)
	Yes	32 (31.4%)	36 (37.9%)	68 (34.5%)
DAY 6	n	103	92	195
	No	74 (71.8%)	64 (69.6%)	138 (70.8%)
	Yes	29 (28.2%)	28 (30.4%)	57 (29.2%)
DAY 7	n	102	91	193
	No	80 (78.4%)	66 (72.5%)	146 (75.6%)
	Yes	22 (21.6%)	25 (27.5%)	47 (24.4%)
DAY 8	n	101	90	191
	No	78 (77.2%)	66 (73.3%)	144 (75.4%)
	Yes	23 (22.8%)	24 (26.7%)	47 (24.6%)
DAY 9	n	100	89	189
	No	78 (78.0%)	68 (76.4%)	146 (77.2%)
	Yes	22 (22.0%)	21 (23.6%)	43 (22.8%)

Note:
Comparaisons at day 3,7,10 and 14 are considered for secondary analysis. The other days are used for exploratory analysis.

CONFIDENTIALITY STATEMENT: Information contained in this statistical report is confidential and should not be disclosed to other parties than those directly involved with the execution of this study without written authorization from Montreal Heart Institute

2 - Efficacy Analysis
2.2 - Secondary Analysis
Table 2.2.3e - Summary of and Statistical Analysis for Presence of Runny Nose at Day 1 to 14, ITT population

		Placebo N=109	Hesperidin N=107	All N=216
DAY 10	n	100	90	190
	No	83 (83.0%)	72 (80.0%)	155 (81.6%)
	Yes	17 (17.0%)	18 (20.0%)	35 (18.4%)
DAY 11	n	98	86	184
	No	83 (84.7%)	71 (82.6%)	154 (83.7%)
	Yes	15 (15.3%)	15 (17.4%)	30 (16.3%)
DAY 12	n	99	85	184
	No	86 (86.9%)	72 (84.7%)	158 (85.9%)
	Yes	13 (13.1%)	13 (15.3%)	26 (14.1%)
DAY 13	n	98	83	181
	No	86 (87.8%)	69 (83.1%)	155 (85.6%)
	Yes	12 (12.2%)	14 (16.9%)	26 (14.4%)
DAY 14	n	95	79	174
	No	85 (89.5%)	67 (84.8%)	152 (87.4%)
	Yes	10 (10.5%)	12 (15.2%)	22 (12.6%)

Note:
Comparaisons at day 3,7,10 and 14 are considered for secondary analysis. The other days are used for exploratory analysis.

CONFIDENTIALITY STATEMENT: Information contained in this statistical report is confidential and should not be disclosed to other parties than those directly involved with the execution of this study without written authorization from Montreal Heart Institute

2 – Efficacy Analysis
2.2 – Secondary Analysis
Table 2.2.3e - Summary of and Statistical Analysis for Presence of Runny Nose at Day 1 to 14, ITT Population (continued)

Generalized linear mixed model (repeated binary logistic regression) with terms for treatment group, time and treatment group x time interaction

Contrast	OR (95% CI)	P-value
Hesperidin vs Placebo at Day 3	1.52 (0.87; 2.67)	0.1438
Hesperidin vs Placebo at Day 7	1.38 (0.71; 2.68)	0.3440
Hesperidin vs Placebo at Day 10	1.22 (0.58; 2.56)	0.5968
Hesperidin vs Placebo at Day 14	1.52 (0.62; 3.76)	0.3620

CONFIDENTIALITY STATEMENT: Information contained in this statistical report is confidential and should not be disclosed to other parties than those directly involved with the execution of this study without written authorization from Montreal Heart Institute

2 - Efficacy Analysis
2.2 - Secondary Analysis
Table 2.2.3f - Summary of and Statistical Analysis for Presence of Shortness of Breath/Difficulty Breathing at Day 1 to 14, ITT population

		Placebo N=109	Hesperidin N=107	All N=216
DAY 1	n	107	105	212
	No	65 (60.7%)	54 (51.4%)	119 (56.1%)
	Yes	42 (39.3%)	51 (48.6%)	93 (43.9%)
DAY 2	n	106	105	211
	No	62 (58.5%)	52 (49.5%)	114 (54.0%)
	Yes	44 (41.5%)	53 (50.5%)	97 (46.0%)
DAY 3	n	103	102	205
	No	68 (66.0%)	56 (54.9%)	124 (60.5%)
	Yes	35 (34.0%)	46 (45.1%)	81 (39.5%)
DAY 4	n	103	96	199
	No	68 (66.0%)	58 (60.4%)	126 (63.3%)
	Yes	35 (34.0%)	38 (39.6%)	73 (36.7%)
DAY 5	n	102	95	197
	No	71 (69.6%)	56 (58.9%)	127 (64.5%)
	Yes	31 (30.4%)	39 (41.1%)	70 (35.5%)
DAY 6	n	103	92	195
	No	76 (73.8%)	56 (60.9%)	132 (67.7%)
	Yes	27 (26.2%)	36 (39.1%)	63 (32.3%)
DAY 7	n	102	91	193
	No	72 (70.6%)	63 (69.2%)	135 (69.9%)
	Yes	30 (29.4%)	28 (30.8%)	58 (30.1%)
DAY 8	n	101	90	191
	No	73 (72.3%)	63 (70.0%)	136 (71.2%)
	Yes	28 (27.7%)	27 (30.0%)	55 (28.8%)
DAY 9	n	100	89	189
	No	79 (79.0%)	64 (71.9%)	143 (75.7%)
	Yes	21 (21.0%)	25 (28.1%)	46 (24.3%)

Note:
Comparaisons at day 3,7,10 and 14 are considered for secondary analysis. The other days are used for exploratory analysis.

CONFIDENTIALITY STATEMENT: Information contained in this statistical report is confidential and should not be disclosed to other parties than those directly involved with the execution of this study without written authorization from Montreal Heart Institute

2 - Efficacy Analysis
2.2 - Secondary Analysis
Table 2.2.3f - Summary of and Statistical Analysis for Presence of Shortness of Breath/Difficulty Breathing at Day 1 to 14, ITT population

		Placebo N=109	Hesperidin N=107	All N=216
DAY 10	n	100	90	190
	No	82 (82.0%)	71 (78.9%)	153 (80.5%)
	Yes	18 (18.0%)	19 (21.1%)	37 (19.5%)
DAY 11	n	98	86	184
	No	80 (81.6%)	70 (81.4%)	150 (81.5%)
	Yes	18 (18.4%)	16 (18.6%)	34 (18.5%)
DAY 12	n	99	85	184
	No	84 (84.8%)	73 (85.9%)	157 (85.3%)
	Yes	15 (15.2%)	12 (14.1%)	27 (14.7%)
DAY 13	n	98	83	181
	No	83 (84.7%)	70 (84.3%)	153 (84.5%)
	Yes	15 (15.3%)	13 (15.7%)	28 (15.5%)
DAY 14	n	95	79	174
	No	82 (86.3%)	65 (82.3%)	147 (84.5%)
	Yes	13 (13.7%)	14 (17.7%)	27 (15.5%)

Note:
Comparaisons at day 3,7,10 and 14 are considered for secondary analysis. The other days are used for exploratory analysis.

CONFIDENTIALITY STATEMENT: Information contained in this statistical report is confidential and should not be disclosed to other parties than those directly involved with the execution of this study without written authorization from Montreal Heart Institute

2 – Efficacy Analysis
2.2 – Secondary Analysis
Table 2.2.3f - Summary of and Statistical Analysis for Presence of Shortness of Breath/Difficulty Breathing at Day 1 to 14, ITT Population (continued)

Generalized linear mixed model (repeated binary logistic regression) with terms for treatment group, time and treatment group x time interaction

Contrast	OR (95% CI)	P-value
Hesperidin vs Placebo at Day 3	1.60 (0.90; 2.82)	0.1068
Hesperidin vs Placebo at Day 7	1.07 (0.57; 1.98)	0.8382
Hesperidin vs Placebo at Day 10	1.22 (0.59; 2.51)	0.5912
Hesperidin vs Placebo at Day 14	1.36 (0.59; 3.11)	0.4677

CONFIDENTIALITY STATEMENT: Information contained in this statistical report is confidential and should not be disclosed to other parties than those directly involved with the execution of this study without written authorization from Montreal Heart Institute

2 – Efficacy Analysis
2.2 – Secondary Analysis
Table 2.2.3g – Summary of and Statistical Analysis for Presence of Nausea/Vomiting at Day 1 to 14, ITT population

		Placebo N=109	Hesperidin N=107	All N=216
DAY 1	n	107	105	212
	No	95 (88.8%)	83 (79.0%)	178 (84.0%)
	Yes	12 (11.2%)	22 (21.0%)	34 (16.0%)
DAY 2	n	106	105	211
	No	91 (85.8%)	83 (79.0%)	174 (82.5%)
	Yes	15 (14.2%)	22 (21.0%)	37 (17.5%)
DAY 3	n	103	102	205
	No	91 (88.3%)	81 (79.4%)	172 (83.9%)
	Yes	12 (11.7%)	21 (20.6%)	33 (16.1%)
DAY 4	n	103	96	199
	No	94 (91.3%)	81 (84.4%)	175 (87.9%)
	Yes	9 (8.7%)	15 (15.6%)	24 (12.1%)
DAY 5	n	102	95	197
	No	95 (93.1%)	80 (84.2%)	175 (88.8%)
	Yes	7 (6.9%)	15 (15.8%)	22 (11.2%)
DAY 6	n	103	92	195
	No	97 (94.2%)	79 (85.9%)	176 (90.3%)
	Yes	6 (5.8%)	13 (14.1%)	19 (9.7%)
DAY 7	n	102	91	193
	No	91 (89.2%)	82 (90.1%)	173 (89.6%)
	Yes	11 (10.8%)	9 (9.9%)	20 (10.4%)
DAY 8	n	101	90	191
	No	95 (94.1%)	81 (90.0%)	176 (92.1%)
	Yes	6 (5.9%)	9 (10.0%)	15 (7.9%)
DAY 9	n	100	89	189
	No	96 (96.0%)	81 (91.0%)	177 (93.7%)
	Yes	4 (4.0%)	8 (9.0%)	12 (6.3%)

Note:
Comparaisons at day 3,7,10 and 14 are considered for secondary analysis. The other days are used for exploratory analysis.

CONFIDENTIALITY STATEMENT: Information contained in this statistical report is confidential and should not be disclosed to other parties than those directly involved with the execution of this study without written authorization from Montreal Heart Institute

2 - Efficacy Analysis
2.2 - Secondary Analysis
Table 2.2.3g - Summary of and Statistical Analysis for Presence of Nausea/Vomiting at Day 1 to 14, ITT population

		Placebo N=109	Hesperidin N=107	All N=216
DAY 10	n	100	90	190
	No	95 (95.0%)	84 (93.3%)	179 (94.2%)
	Yes	5 (5.0%)	6 (6.7%)	11 (5.8%)
DAY 11	n	98	86	184
	No	93 (94.9%)	82 (95.3%)	175 (95.1%)
	Yes	5 (5.1%)	4 (4.7%)	9 (4.9%)
DAY 12	n	99	85	184
	No	96 (97.0%)	83 (97.6%)	179 (97.3%)
	Yes	3 (3.0%)	2 (2.4%)	5 (2.7%)
DAY 13	n	98	83	181
	No	94 (95.9%)	82 (98.8%)	176 (97.2%)
	Yes	4 (4.1%)	1 (1.2%)	5 (2.8%)
DAY 14	n	95	79	174
	No	93 (97.9%)	77 (97.5%)	170 (97.7%)
	Yes	2 (2.1%)	2 (2.5%)	4 (2.3%)

Note:
Comparaisons at day 3,7,10 and 14 are considered for secondary analysis. The other days are used for exploratory analysis.

CONFIDENTIALITY STATEMENT: Information contained in this statistical report is confidential and should not be disclosed to other parties than those directly involved with the execution of this study without written authorization from Montreal Heart Institute

2 – Efficacy Analysis
2.2 – Secondary Analysis
Table 2.2.3g - Summary of and Statistical Analysis for Presence of Nausea/Vomiting at Day 1 to 14, ITT Population (continued)

Generalized linear mixed model (repeated binary logistic regression) with terms for treatment group, time and treatment group x time interaction

Contrast	OR (95% CI)	P-value
Hesperidin vs Placebo at Day 3	1.97 (0.91; 4.27)	0.0875
Hesperidin vs Placebo at Day 7	0.91 (0.36; 2.32)	0.8397
Hesperidin vs Placebo at Day 10	1.36 (0.40; 4.65)	0.6265
Hesperidin vs Placebo at Day 14	1.21 (0.16; 8.91)	0.8528

CONFIDENTIALITY STATEMENT: Information contained in this statistical report is confidential and should not be disclosed to other parties than those directly involved with the execution of this study without written authorization from Montreal Heart Institute

2 - Efficacy Analysis
2.2 - Secondary Analysis
Table 2.2.3h - Summary of and Statistical Analysis for Presence of Headache at Day 1 to 14, ITT population

		Placebo N=109	Hesperidin N=107	All N=216
DAY 1	n	107	105	212
	No	39 (36.4%)	38 (36.2%)	77 (36.3%)
	Yes	68 (63.6%)	67 (63.8%)	135 (63.7%)
DAY 2	n	106	105	211
	No	54 (50.9%)	49 (46.7%)	103 (48.8%)
	Yes	52 (49.1%)	56 (53.3%)	108 (51.2%)
DAY 3	n	103	102	205
	No	62 (60.2%)	54 (52.9%)	116 (56.6%)
	Yes	41 (39.8%)	48 (47.1%)	89 (43.4%)
DAY 4	n	103	96	199
	No	68 (66.0%)	53 (55.2%)	121 (60.8%)
	Yes	35 (34.0%)	43 (44.8%)	78 (39.2%)
DAY 5	n	102	95	197
	No	66 (64.7%)	58 (61.1%)	124 (62.9%)
	Yes	36 (35.3%)	37 (38.9%)	73 (37.1%)
DAY 6	n	103	92	195
	No	73 (70.9%)	58 (63.0%)	131 (67.2%)
	Yes	30 (29.1%)	34 (37.0%)	64 (32.8%)
DAY 7	n	102	91	193
	No	68 (66.7%)	57 (62.6%)	125 (64.8%)
	Yes	34 (33.3%)	34 (37.4%)	68 (35.2%)
DAY 8	n	101	90	191
	No	74 (73.3%)	68 (75.6%)	142 (74.3%)
	Yes	27 (26.7%)	22 (24.4%)	49 (25.7%)
DAY 9	n	100	89	189
	No	79 (79.0%)	70 (78.7%)	149 (78.8%)
	Yes	21 (21.0%)	19 (21.3%)	40 (21.2%)

Note:
Comparaisons at day 3,7,10 and 14 are considered for secondary analysis. The other days are used for exploratory analysis.

CONFIDENTIALITY STATEMENT: Information contained in this statistical report is confidential and should not be disclosed to other parties than those directly involved with the execution of this study without written authorization from Montreal Heart Institute

2 - Efficacy Analysis
2.2 - Secondary Analysis
Table 2.2.3h - Summary of and Statistical Analysis for Presence of Headache at Day 1 to 14, ITT population

		Placebo N=109	Hesperidin N=107	All N=216
DAY 10	n	100	90	190
	No	80 (80.0%)	68 (75.6%)	148 (77.9%)
	Yes	20 (20.0%)	22 (24.4%)	42 (22.1%)
DAY 11	n	98	86	184
	No	81 (82.7%)	70 (81.4%)	151 (82.1%)
	Yes	17 (17.3%)	16 (18.6%)	33 (17.9%)
DAY 12	n	99	85	184
	No	82 (82.8%)	73 (85.9%)	155 (84.2%)
	Yes	17 (17.2%)	12 (14.1%)	29 (15.8%)
DAY 13	n	98	83	181
	No	84 (85.7%)	74 (89.2%)	158 (87.3%)
	Yes	14 (14.3%)	9 (10.8%)	23 (12.7%)
DAY 14	n	95	79	174
	No	86 (90.5%)	69 (87.3%)	155 (89.1%)
	Yes	9 (9.5%)	10 (12.7%)	19 (10.9%)

Note:
Comparaisons at day 3,7,10 and 14 are considered for secondary analysis. The other days are used for exploratory analysis.

CONFIDENTIALITY STATEMENT: Information contained in this statistical report is confidential and should not be disclosed to other parties than those directly involved with the execution of this study without written authorization from Montreal Heart Institute

2 – Efficacy Analysis
2.2 – Secondary Analysis
Table 2.2.3h - Summary of and Statistical Analysis for Presence of Headache at Day 1 to 14, ITT Population (continued)

Generalized linear mixed model (repeated binary logistic regression) with terms for treatment group, time and treatment group x time interaction

Contrast	OR (95% CI)	P-value
Hesperidin vs Placebo at Day 3	1.34 (0.77; 2.35)	0.2983
Hesperidin vs Placebo at Day 7	1.19 (0.66; 2.16)	0.5611
Hesperidin vs Placebo at Day 10	1.29 (0.65; 2.58)	0.4643
Hesperidin vs Placebo at Day 14	1.38 (0.53; 3.62)	0.5063

CONFIDENTIALITY STATEMENT: Information contained in this statistical report is confidential and should not be disclosed to other parties than those directly involved with the execution of this study without written authorization from Montreal Heart Institute

2 - Efficacy Analysis
2.2 - Secondary Analysis
Table 2.2.3i - Summary of and Statistical Analysis for Presence of General Weakness at Day 1 to 14, ITT population

		Placebo N=109	Hesperidin N=107	All N=216
DAY 1	n	107	105	212
	No	40 (37.4%)	32 (30.5%)	72 (34.0%)
	Yes	67 (62.6%)	73 (69.5%)	140 (66.0%)
DAY 2	n	106	105	211
	No	45 (42.5%)	35 (33.3%)	80 (37.9%)
	Yes	61 (57.5%)	70 (66.7%)	131 (62.1%)
DAY 3	n	103	102	205
	No	48 (46.6%)	39 (38.2%)	87 (42.4%)
	Yes	55 (53.4%)	63 (61.8%)	118 (57.6%)
DAY 4	n	103	96	199
	No	49 (47.6%)	37 (38.5%)	86 (43.2%)
	Yes	54 (52.4%)	59 (61.5%)	113 (56.8%)
DAY 5	n	102	95	197
	No	53 (52.0%)	41 (43.2%)	94 (47.7%)
	Yes	49 (48.0%)	54 (56.8%)	103 (52.3%)
DAY 6	n	103	92	195
	No	62 (60.2%)	47 (51.1%)	109 (55.9%)
	Yes	41 (39.8%)	45 (48.9%)	86 (44.1%)
DAY 7	n	102	91	193
	No	62 (60.8%)	52 (57.1%)	114 (59.1%)
	Yes	40 (39.2%)	39 (42.9%)	79 (40.9%)
DAY 8	n	101	90	191
	No	69 (68.3%)	56 (62.2%)	125 (65.4%)
	Yes	32 (31.7%)	34 (37.8%)	66 (34.6%)
DAY 9	n	100	89	189
	No	73 (73.0%)	62 (69.7%)	135 (71.4%)
	Yes	27 (27.0%)	27 (30.3%)	54 (28.6%)

Note:
Comparaisons at day 3,7,10 and 14 are considered for secondary analysis. The other days are used for exploratory analysis.

CONFIDENTIALITY STATEMENT: Information contained in this statistical report is confidential and should not be disclosed to other parties than those directly involved with the execution of this study without written authorization from Montreal Heart Institute

2 - Efficacy Analysis
2.2 - Secondary Analysis
Table 2.2.3i - Summary of and Statistical Analysis for Presence of General Weakness at Day 1 to 14, ITT population

		Placebo N=109	Hesperidin N=107	All N=216
DAY 10	n	100	90	190
	No	79 (79.0%)	70 (77.8%)	149 (78.4%)
	Yes	21 (21.0%)	20 (22.2%)	41 (21.6%)
DAY 11	n	98	86	184
	No	78 (79.6%)	67 (77.9%)	145 (78.8%)
	Yes	20 (20.4%)	19 (22.1%)	39 (21.2%)
DAY 12	n	99	85	184
	No	78 (78.8%)	69 (81.2%)	147 (79.9%)
	Yes	21 (21.2%)	16 (18.8%)	37 (20.1%)
DAY 13	n	98	83	181
	No	78 (79.6%)	68 (81.9%)	146 (80.7%)
	Yes	20 (20.4%)	15 (18.1%)	35 (19.3%)
DAY 14	n	95	79	174
	No	78 (82.1%)	65 (82.3%)	143 (82.2%)
	Yes	17 (17.9%)	14 (17.7%)	31 (17.8%)

Note:
Comparaisons at day 3,7,10 and 14 are considered for secondary analysis. The other days are used for exploratory analysis.

CONFIDENTIALITY STATEMENT: Information contained in this statistical report is confidential and should not be disclosed to other parties than those directly involved with the execution of this study without written authorization from Montreal Heart Institute

2 – Efficacy Analysis
2.2 – Secondary Analysis
Table 2.2.3i - Summary of and Statistical Analysis for Presence of General Weakness at Day 1 to 14, ITT Population (continued)

Generalized linear mixed model (repeated binary logistic regression) with terms for treatment group, time and treatment group x time interaction

Contrast	OR (95% CI)	P-value
Hesperidin vs Placebo at Day 3	1.41 (0.80; 2.47)	0.2292
Hesperidin vs Placebo at Day 7	1.16 (0.65; 2.07)	0.6097
Hesperidin vs Placebo at Day 10	1.07 (0.54; 2.16)	0.8389
Hesperidin vs Placebo at Day 14	0.99 (0.45; 2.17)	0.9764

CONFIDENTIALITY STATEMENT: Information contained in this statistical report is confidential and should not be disclosed to other parties than those directly involved with the execution of this study without written authorization from Montreal Heart Institute

2 - Efficacy Analysis
2.2 - Secondary Analysis
Table 2.2.3j - Summary of and Statistical Analysis for Presence of Pain at Day 1 to 14, ITT population

		Placebo N=109	Hesperidin N=107	All N=216
DAY 1	n	107	105	212
	No	44 (41.1%)	43 (41.0%)	87 (41.0%)
	Yes	63 (58.9%)	62 (59.0%)	125 (59.0%)
DAY 2	n	106	105	211
	No	55 (51.9%)	47 (44.8%)	102 (48.3%)
	Yes	51 (48.1%)	58 (55.2%)	109 (51.7%)
DAY 3	n	103	102	205
	No	53 (51.5%)	58 (56.9%)	111 (54.1%)
	Yes	50 (48.5%)	44 (43.1%)	94 (45.9%)
DAY 4	n	103	96	199
	No	65 (63.1%)	63 (65.6%)	128 (64.3%)
	Yes	38 (36.9%)	33 (34.4%)	71 (35.7%)
DAY 5	n	102	95	197
	No	69 (67.6%)	68 (71.6%)	137 (69.5%)
	Yes	33 (32.4%)	27 (28.4%)	60 (30.5%)
DAY 6	n	103	92	195
	No	79 (76.7%)	71 (77.2%)	150 (76.9%)
	Yes	24 (23.3%)	21 (22.8%)	45 (23.1%)
DAY 7	n	102	91	193
	No	79 (77.5%)	74 (81.3%)	153 (79.3%)
	Yes	23 (22.5%)	17 (18.7%)	40 (20.7%)
DAY 8	n	101	90	191
	No	82 (81.2%)	77 (85.6%)	159 (83.2%)
	Yes	19 (18.8%)	13 (14.4%)	32 (16.8%)
DAY 9	n	100	89	189
	No	84 (84.0%)	76 (85.4%)	160 (84.7%)
	Yes	16 (16.0%)	13 (14.6%)	29 (15.3%)

Note:
Comparaisons at day 3,7,10 and 14 are considered for secondary analysis. The other days are used for exploratory analysis.

CONFIDENTIALITY STATEMENT: Information contained in this statistical report is confidential and should not be disclosed to other parties than those directly involved with the execution of this study without written authorization from Montreal Heart Institute

2 - Efficacy Analysis
2.2 - Secondary Analysis
Table 2.2.3j - Summary of and Statistical Analysis for Presence of Pain at Day 1 to 14, ITT population

		Placebo N=109	Hesperidin N=107	All N=216
DAY 10	n	100	90	190
	No	86 (86.0%)	77 (85.6%)	163 (85.8%)
	Yes	14 (14.0%)	13 (14.4%)	27 (14.2%)
DAY 11	n	98	86	184
	No	86 (87.8%)	77 (89.5%)	163 (88.6%)
	Yes	12 (12.2%)	9 (10.5%)	21 (11.4%)
DAY 12	n	99	85	184
	No	89 (89.9%)	79 (92.9%)	168 (91.3%)
	Yes	10 (10.1%)	6 (7.1%)	16 (8.7%)
DAY 13	n	98	83	181
	No	89 (90.8%)	78 (94.0%)	167 (92.3%)
	Yes	9 (9.2%)	5 (6.0%)	14 (7.7%)
DAY 14	n	95	79	174
	No	87 (91.6%)	74 (93.7%)	161 (92.5%)
	Yes	8 (8.4%)	5 (6.3%)	13 (7.5%)

Note:
Comparaisons at day 3,7,10 and 14 are considered for secondary analysis. The other days are used for exploratory analysis.

CONFIDENTIALITY STATEMENT: Information contained in this statistical report is confidential and should not be disclosed to other parties than those directly involved with the execution of this study without written authorization from Montreal Heart Institute

2 – Efficacy Analysis
2.2 – Secondary Analysis
Table 2.2.3j - Summary of and Statistical Analysis for Presence of Pain at Day 1 to 14, ITT Population (continued)

Generalized linear mixed model (repeated binary logistic regression) with terms for treatment group, time and treatment group x time interaction

Contrast	OR (95% CI)	P-value
Hesperidin vs Placebo at Day 3	0.80 (0.46; 1.40)	0.4403
Hesperidin vs Placebo at Day 7	0.79 (0.39; 1.60)	0.5112
Hesperidin vs Placebo at Day 10	1.04 (0.46; 2.36)	0.9306
Hesperidin vs Placebo at Day 14	0.73 (0.23; 2.36)	0.6046

CONFIDENTIALITY STATEMENT: Information contained in this statistical report is confidential and should not be disclosed to other parties than those directly involved with the execution of this study without written authorization from Montreal Heart Institute

2 - Efficacy Analysis
2.2 - Secondary Analysis
Table 2.2.3k - Summary of and Statistical Analysis for Presence of Irritability/Confusion at Day 1 to 14, ITT population

		Placebo N=109	Hesperidin N=107	All N=216
DAY 1	n	107	105	212
	No	79 (73.8%)	80 (76.2%)	159 (75.0%)
	Yes	28 (26.2%)	25 (23.8%)	53 (25.0%)
DAY 2	n	106	105	211
	No	80 (75.5%)	83 (79.0%)	163 (77.3%)
	Yes	26 (24.5%)	22 (21.0%)	48 (22.7%)
DAY 3	n	103	102	205
	No	85 (82.5%)	82 (80.4%)	167 (81.5%)
	Yes	18 (17.5%)	20 (19.6%)	38 (18.5%)
DAY 4	n	103	96	199
	No	83 (80.6%)	77 (80.2%)	160 (80.4%)
	Yes	20 (19.4%)	19 (19.8%)	39 (19.6%)
DAY 5	n	102	95	197
	No	87 (85.3%)	85 (89.5%)	172 (87.3%)
	Yes	15 (14.7%)	10 (10.5%)	25 (12.7%)
DAY 6	n	103	92	195
	No	91 (88.3%)	81 (88.0%)	172 (88.2%)
	Yes	12 (11.7%)	11 (12.0%)	23 (11.8%)
DAY 7	n	102	91	193
	No	92 (90.2%)	85 (93.4%)	177 (91.7%)
	Yes	10 (9.8%)	6 (6.6%)	16 (8.3%)
DAY 8	n	101	90	191
	No	92 (91.1%)	85 (94.4%)	177 (92.7%)
	Yes	9 (8.9%)	5 (5.6%)	14 (7.3%)
DAY 9	n	100	89	189
	No	94 (94.0%)	84 (94.4%)	178 (94.2%)
	Yes	6 (6.0%)	5 (5.6%)	11 (5.8%)

Note:
Comparaisons at day 3,7,10 and 14 are considered for secondary analysis. The other days are used for exploratory analysis.

CONFIDENTIALITY STATEMENT: Information contained in this statistical report is confidential and should not be disclosed to other parties than those directly involved with the execution of this study without written authorization from Montreal Heart Institute

2 - Efficacy Analysis
2.2 - Secondary Analysis
Table 2.2.3k - Summary of and Statistical Analysis for Presence of Irritability/Confusion at Day 1 to 14, ITT population

		Placebo N=109	Hesperidin N=107	All N=216
DAY 10	n	100	90	190
	No	96 (96.0%)	86 (95.6%)	182 (95.8%)
	Yes	4 (4.0%)	4 (4.4%)	8 (4.2%)
DAY 11	n	98	86	184
	No	93 (94.9%)	82 (95.3%)	175 (95.1%)
	Yes	5 (5.1%)	4 (4.7%)	9 (4.9%)
DAY 12	n	99	85	184
	No	97 (98.0%)	82 (96.5%)	179 (97.3%)
	Yes	2 (2.0%)	3 (3.5%)	5 (2.7%)
DAY 13	n	98	83	181
	No	96 (98.0%)	80 (96.4%)	176 (97.2%)
	Yes	2 (2.0%)	3 (3.6%)	5 (2.8%)
DAY 14	n	95	79	174
	No	94 (98.9%)	76 (96.2%)	170 (97.7%)
	Yes	1 (1.1%)	3 (3.8%)	4 (2.3%)

Note:
Comparaisons at day 3,7,10 and 14 are considered for secondary analysis. The other days are used for exploratory analysis.

CONFIDENTIALITY STATEMENT: Information contained in this statistical report is confidential and should not be disclosed to other parties than those directly involved with the execution of this study without written authorization from Montreal Heart Institute

2 – Efficacy Analysis
2.2 – Secondary Analysis
Table 2.2.3k - Summary of and Statistical Analysis for Presence of Irritability/Confusion at Day 1 to 14, ITT Population (continued)

Generalized linear mixed model (repeated binary logistic regression) with terms for treatment group, time and treatment group x time interaction

Contrast	OR (95% CI)	P-value
Hesperidin vs Placebo at Day 3	1.15 (0.57; 2.34)	0.6963
Hesperidin vs Placebo at Day 7	0.65 (0.22; 1.88)	0.4250
Hesperidin vs Placebo at Day 10	1.12 (0.27; 4.65)	0.8797
Hesperidin vs Placebo at Day 14	3.71 (0.37; 37.03)	0.2634

CONFIDENTIALITY STATEMENT: Information contained in this statistical report is confidential and should not be disclosed to other parties than those directly involved with the execution of this study without written authorization from Montreal Heart Institute

2 - Efficacy Analysis
2.2 - Secondary Analysis
Table 2.2.31 - Summary of and Statistical Analysis for Presence of Diarrhea at Day 1 to 14, ITT population

		Placebo N=109	Hesperidin N=107	All N=216
DAY 1	n	107	105	212
	No	85 (79.4%)	82 (78.1%)	167 (78.8%)
	Yes	22 (20.6%)	23 (21.9%)	45 (21.2%)
DAY 2	n	106	105	211
	No	84 (79.2%)	83 (79.0%)	167 (79.1%)
	Yes	22 (20.8%)	22 (21.0%)	44 (20.9%)
DAY 3	n	103	102	205
	No	87 (84.5%)	80 (78.4%)	167 (81.5%)
	Yes	16 (15.5%)	22 (21.6%)	38 (18.5%)
DAY 4	n	103	96	199
	No	90 (87.4%)	72 (75.0%)	162 (81.4%)
	Yes	13 (12.6%)	24 (25.0%)	37 (18.6%)
DAY 5	n	102	95	197
	No	89 (87.3%)	76 (80.0%)	165 (83.8%)
	Yes	13 (12.7%)	19 (20.0%)	32 (16.2%)
DAY 6	n	103	92	195
	No	92 (89.3%)	72 (78.3%)	164 (84.1%)
	Yes	11 (10.7%)	20 (21.7%)	31 (15.9%)
DAY 7	n	102	91	193
	No	92 (90.2%)	76 (83.5%)	168 (87.0%)
	Yes	10 (9.8%)	15 (16.5%)	25 (13.0%)
DAY 8	n	101	90	191
	No	92 (91.1%)	77 (85.6%)	169 (88.5%)
	Yes	9 (8.9%)	13 (14.4%)	22 (11.5%)
DAY 9	n	100	89	189
	No	92 (92.0%)	81 (91.0%)	173 (91.5%)
	Yes	8 (8.0%)	8 (9.0%)	16 (8.5%)

Note:
Comparaisons at day 3,7,10 and 14 are considered for secondary analysis. The other days are used for exploratory analysis.

CONFIDENTIALITY STATEMENT: Information contained in this statistical report is confidential and should not be disclosed to other parties than those directly involved with the execution of this study without written authorization from Montreal Heart Institute

2 - Efficacy Analysis
2.2 - Secondary Analysis
Table 2.2.31 - Summary of and Statistical Analysis for Presence of Diarrhea at Day 1 to 14, ITT population

		Placebo N=109	Hesperidin N=107	All N=216
DAY 10	n	100	90	190
	No	94 (94.0%)	84 (93.3%)	178 (93.7%)
	Yes	6 (6.0%)	6 (6.7%)	12 (6.3%)
DAY 11	n	98	86	184
	No	94 (95.9%)	79 (91.9%)	173 (94.0%)
	Yes	4 (4.1%)	7 (8.1%)	11 (6.0%)
DAY 12	n	99	85	184
	No	96 (97.0%)	77 (90.6%)	173 (94.0%)
	Yes	3 (3.0%)	8 (9.4%)	11 (6.0%)
DAY 13	n	98	83	181
	No	94 (95.9%)	76 (91.6%)	170 (93.9%)
	Yes	4 (4.1%)	7 (8.4%)	11 (6.1%)
DAY 14	n	95	79	174
	No	91 (95.8%)	75 (94.9%)	166 (95.4%)
	Yes	4 (4.2%)	4 (5.1%)	8 (4.6%)

Note:
Comparaisons at day 3,7,10 and 14 are considered for secondary analysis. The other days are used for exploratory analysis.

CONFIDENTIALITY STATEMENT: Information contained in this statistical report is confidential and should not be disclosed to other parties than those directly involved with the execution of this study without written authorization from Montreal Heart Institute

2 – Efficacy Analysis
2.2 – Secondary Analysis
Table 2.2.31 - Summary of and Statistical Analysis for Presence of Diarrhea at Day 1 to 14, ITT Population (continued)

Generalized linear mixed model (repeated binary logistic regression) with terms for treatment group, time and treatment group x time interaction

Contrast	OR (95% CI)	P-value
Hesperidin vs Placebo at Day 3	1.50 (0.73; 3.06)	0.2710
Hesperidin vs Placebo at Day 7	1.82 (0.77; 4.30)	0.1748
Hesperidin vs Placebo at Day 10	1.12 (0.34; 3.63)	0.8513
Hesperidin vs Placebo at Day 14	1.21 (0.29; 5.07)	0.7906

CONFIDENTIALITY STATEMENT: Information contained in this statistical report is confidential and should not be disclosed to other parties than those directly involved with the execution of this study without written authorization from Montreal Heart Institute

2 - Efficacy Analysis
2.2 - Secondary Analysis
Table 2.2.3m - Summary of and Statistical Analysis for Presence of Anosmia at Day 1 to 14, ITT Population

		Placebo N=109	Hesperidin N=107	All N=216
DAY 1	n	107	105	212
	No	57 (53.3%)	62 (59.0%)	119 (56.1%)
	Yes	50 (46.7%)	43 (41.0%)	93 (43.9%)
DAY 2	n	106	105	211
	No	52 (49.1%)	56 (53.3%)	108 (51.2%)
	Yes	54 (50.9%)	49 (46.7%)	103 (48.8%)
DAY 3	n	103	102	205
	No	44 (42.7%)	50 (49.0%)	94 (45.9%)
	Yes	59 (57.3%)	52 (51.0%)	111 (54.1%)
DAY 4	n	103	96	199
	No	44 (42.7%)	48 (50.0%)	92 (46.2%)
	Yes	59 (57.3%)	48 (50.0%)	107 (53.8%)
DAY 5	n	102	95	197
	No	45 (44.1%)	45 (47.4%)	90 (45.7%)
	Yes	57 (55.9%)	50 (52.6%)	107 (54.3%)
DAY 6	n	103	92	195
	No	52 (50.5%)	43 (46.7%)	95 (48.7%)
	Yes	51 (49.5%)	49 (53.3%)	100 (51.3%)
DAY 7	n	102	91	193
	No	55 (53.9%)	48 (52.7%)	103 (53.4%)
	Yes	47 (46.1%)	43 (47.3%)	90 (46.6%)
DAY 8	n	101	90	191
	No	60 (59.4%)	50 (55.6%)	110 (57.6%)
	Yes	41 (40.6%)	40 (44.4%)	81 (42.4%)
DAY 9	n	100	89	189
	No	61 (61.0%)	51 (57.3%)	112 (59.3%)
	Yes	39 (39.0%)	38 (42.7%)	77 (40.7%)

Note:
Comparaisons at day 3,7,10 and 14 are considered for secondary analysis. The other days are used for exploratory analysis.

CONFIDENTIALITY STATEMENT: Information contained in this statistical report is confidential and should not be disclosed to other parties than those directly involved with the execution of this study without written authorization from Montreal Heart Institute

2 - Efficacy Analysis
2.2 - Secondary Analysis
Table 2.2.3m - Summary of and Statistical Analysis for Presence of Anosmia at Day 1 to 14, ITT Population

		Placebo N=109	Hesperidin N=107	All N=216
DAY 10	n	100	90	190
	No	63 (63.0%)	56 (62.2%)	119 (62.6%)
	Yes	37 (37.0%)	34 (37.8%)	71 (37.4%)
DAY 11	n	98	86	184
	No	63 (64.3%)	55 (64.0%)	118 (64.1%)
	Yes	35 (35.7%)	31 (36.0%)	66 (35.9%)
DAY 12	n	99	85	184
	No	65 (65.7%)	58 (68.2%)	123 (66.8%)
	Yes	34 (34.3%)	27 (31.8%)	61 (33.2%)
DAY 13	n	98	83	181
	No	65 (66.3%)	58 (69.9%)	123 (68.0%)
	Yes	33 (33.7%)	25 (30.1%)	58 (32.0%)
DAY 14	n	95	79	174
	No	64 (67.4%)	59 (74.7%)	123 (70.7%)
	Yes	31 (32.6%)	20 (25.3%)	51 (29.3%)

Note:
Comparaisons at day 3,7,10 and 14 are considered for secondary analysis. The other days are used for exploratory analysis.

CONFIDENTIALITY STATEMENT: Information contained in this statistical report is confidential and should not be disclosed to other parties than those directly involved with the execution of this study without written authorization from Montreal Heart Institute

2 – Efficacy Analysis
2.2 – Secondary Analysis
Table 2.2.3m - Summary of and Statistical Analysis for Presence of Anosmia at Day 1 to 14, ITT Population (continued)

Generalized linear mixed model (repeated binary logistic regression) with terms for treatment group, time and treatment group x time interaction

Contrast	OR (95% CI)	P-value
Hesperidin vs Placebo at Day 3	0.78 (0.45; 1.35)	0.3686
Hesperidin vs Placebo at Day 7	1.05 (0.59; 1.86)	0.8711
Hesperidin vs Placebo at Day 10	1.03 (0.57; 1.87)	0.9124
Hesperidin vs Placebo at Day 14	0.70 (0.36; 1.37)	0.2952

CONFIDENTIALITY STATEMENT: Information contained in this statistical report is confidential and should not be disclosed to other parties than those directly involved with the execution of this study without written authorization from Montreal Heart Institute
