# Supplementary material for: Fourteen-Day Evolution of COVID-19 Symptoms during the Third Wave in Nonvaccinated Subjects and Effects of Hesperidin Therapy: A Randomized, Double-Blinded, Placebo-Controlled Study
Source: Evid Based Complement Alternat Med. 2022 Nov 3;2022:3125662. doi: 10.1155/2022/3125662 (PMC9649310; doi:10.1155/2022/3125662)
Supplement: Supplementary Materials — S1 Protocol. Version 2.0 of the Hesperidin study protocol approved by the Montreal Heart Institute Research and Ethics Committees. S2 table. Proportion of patients with group A COVID-19 symptoms,attrition bias analysis. S3 table. Summary and statistical analysis for the presence of each 13 COVID-19 symptoms. S4 table. Overall summary of treatment emergent adverse events (TEAEs) and serious adverse events (TESAEs). [file 3125662.f1.zip › S4 Table.docx]

**S4 Table. Overall summary of treatment emergent adverse events (TEAE) and serious adverse events (TESAE), safety population.**

|  | **Placebo**  **N = 108** | **Hesperidin**  **N = 107** | **All**  **N = 215** |
| --- | --- | --- | --- |
| **Total number of TEAEs reported** | 16 | 23 | 39 |
| **Subjects with at least one TEAE** | 16 (14.8%) | 20 (18.7%) | 36 (16.7%) |
| **Subjects with at least one severe TEAE** | 2 (1.9%) | 3 (2.8%) | 5 (2.3%) |
| **Subjects with at least one TEAE related to the study treatment** | 4 (3.7%) | 3 (2.8%) | 7 (3.3%) |
| **Subjects with at least one TEAE leading to drug withdrawal** | 5 (4.6%) | 8 (7.5%) | 13 (6.0%) |
|  |  |  |  |
| **Total number of TESAEs reported** | 1 | 5 | 6 |
| **Subjects with at least one TESAE** | 1 (0.9%) | 4 (3.7%) | 5 (2.3%) |
| **Subjects with at least one severe TESAE** | 1 (0.9%) | 3 (2.8%) | 4 (1.9%) |
| **Subjects with at least one TESAE related to the study treatment** | 0 (0%) | 0 (0%) | 0 (0%) |
| **Subjects with at least one TEAE leading to drug withdrawal** | 1 (0.9%) | 2 (1.9%) | 3 (1.4%) |
